# Altered Sertoli Cell Function Contributes to Spermatogenic Arrest in Dogs with Chronic Asymptomatic Orchitis

**DOI:** 10.3390/ijms26031108

**Published:** 2025-01-27

**Authors:** Pauline Rehder, Eva-Maria Packeiser, Hanna Körber, Sandra Goericke-Pesch

**Affiliations:** Reproductive Unit—Clinic for Small Animals, University of Veterinary Medicine Hannover, Foundation, 30559 Hannover, Germany; pauline.rehder@tiho-hannover.de (P.R.); eva-maria.packeiser@tiho-hannover.de (E.-M.P.); hanna.koerber@tiho-hannover.de (H.K.)

**Keywords:** bFGF, BMP4, CXCL12, GDNF, LDHC, WNT5A, azoospermia, chronic orchitis, canine testis, Sertoli cells

## Abstract

Acquired infertility due to chronic asymptomatic orchitis (CAO) is a common finding in male dogs. It is characterized by spermatogenic arrest, a significant reduction in spermatogonia, immune cell infiltration and a disruption of the blood–testis barrier. Sertoli cells are a key factor for spermatogenesis and the testicular micromilieu. We hypothesize altered Sertoli cell function to be involved in the pathogenesis of canine CAO. Consequently, the aim was to gain further insights into the spermatogonial stem cell niche and Sertoli cell function in CAO-affected dogs. Therefore, the testicular expression of the Sertoli cell-derived factors *bFGF*, *GDNF*, *WNT5A*, *BMP4*, *CXCL12* and *LDHC* were evaluated in 15 CAO testis tissues and 10 normospermic controls by relative quantitative real-time PCR (qPCR). Additionally, the protein expression patterns of bFGF, GDNF and WNT5A were visualized immunohistochemically (IHC). This study revealed an overexpression of bFGF (IHC, *p* < 0.0001), *GDNF* (qPCR, *p* = 0.0036), WNT5A (IHC, *p* = 0.0066) and *CXCL12* (qPCR, *p* = 0.0003) and a reduction in *BMP4* (qPCR, *p* = 0.0041) and *LDHC* (qPCR, *p* = 0.0003) in CAO-affected testis in dogs, clearly confirming impaired Sertoli cell function in canine CAO. Sertoli cell function is essential for spermatogenesis and must be considered for potential therapeutic approaches.

## 1. Introduction

If a previously fertile stud dog suddenly fails to mate successfully, the owners are presented with an emotional and financial burden, and their veterinarian faces a complex problem. Additionally, the causative agent of infertility often cannot be identified, therapy is impossible, and the male dog remains irreversibly infertile. Non-obstructive azoospermia (NOA) is the most common finding in infertile, andrologically healthy male dogs [1,2]. In humans, NOA affects about 1% of the male population and 10% of infertile men [3,4,5]. In a larger group of male dogs with NOA, we were able to identify chronic asymptomatic orchitis (CAO) as its main cause in dogs [6]. Prominent histopathological changes including a spermatogenic arrest [6], a significant reduction in spermatogonia [7,8], immune cell infiltration [9] and a disrupted blood–testis barrier (BTB) [10] are typical characteristics of canine CAO. This, together with downregulation of the androgen receptor [11], suggests functional and hormonal disruptions contributing to the pathogenesis of CAO, as well as involvement of Sertoli cells as a crucial part of the BTB.

Sertoli cells have a special significance for physiological spermatogenesis, which has been undisputed since their first description in 1885 [12,13]. Apart from minor differences during testis development, the morphology and function of post-pubertal, mature Sertoli cells are highly conserved among mammals [14]. The length of the Sertoli cells extends from the basal membrane of the seminiferous tubules towards the lumen, and they surround the different generations of germ cells with their cytoplasmic extensions, supporting the testicular tissue structurally and functionally in their various functions, forming the BTB, nourishing the germ cells, performing phagocytosis during cell degeneration and releasing the spermatids [13,15]. Moreover, in humans and other mammalians, they secrete various factors and are thus also part of the complex spermatogonial stem cell (SSC) niche [16]. In the final stage of CAO, after elimination of all germ cells, only Sertoli cells remain, possibly due to the expression of anti-apoptotic factors [17]. Significant upregulation of PTGS2 (prostaglandin endoperoxidase 2), the key enzyme of inflammation, in Sertoli cells of CAO-affected dogs [18] leads to the assumption that impaired or modified SC function plays an important role in the development and/or maintenance of CAO.

The regulation of the maintenance, self-renewal and differentiation of the SSCs is essential for normal spermatogenesis in mice [19]. As comprehensively learned from mouse, rat and cell culture, the complex paracrine, molecular and cellular interactions between SSCs and associated cells, such as Sertoli cells and other somatic cells [20], which together form the SSC niche, play a crucial role in the regulation of spermatogenesis [21]. Several functional, Sertoli cell-derived factors are involved in the complex SSC niche crosstalk (Table 1).

We postulate altered Sertoli cell function to be involved in canine CAO and therefore investigated functional Sertoli cell markers in CAO-affected dog testes and healthy controls via relative quantitative real-time PCR (qPCR) at the mRNA level and immunohistochemically at the protein level. Sertoli cell-secreted factors such as GDNF [26], but also bFGF, CXCL12 and WNT5A, contribute to the SSC niche regulation in the mouse testis [20,21,23]. GDNF, bFGF and CXCL12 are closely linked factors, without the totality of which no maintenance of the SSC population is possible [32,37,38]. On the other hand, the growth factor WNT5A and BMP4 regulate Sertoli cell proliferation and SSC self-renewal [28,29,30]. Interestingly, BMP4 is downregulated in NOA-affected men [31]. Zhao et al. [39] postulated immature Sertoli cells in human idiopathic NOA. Knowing that the maturation state is altered in canine CAO (unpublished data), we additionally included WNT5A and LDHC in our analysis.

Detailed characterization of canine CAO seems also especially necessary as it might serve as a suitable model for human CAO [40,41], in addition to the well-studied experimentally induced autoimmune orchitis rodent model [42,43]. With a longer lifespan than rodents, the dog was previously shown to be a suitable sentinel species for environmental effects on human fertility [44,45] and is a highly appreciated model species in ageing [46] and oncological research [47]. Interestingly, both human and canine CAO are characterized by similar clinical and histological features [6,9,40,41]. Potentially, as they share our living conditions, lifestyle and environmental influences, dogs might help to identify inducing or promoting factors of CAO.

While Reifarth et al. demonstrated resilient SSC in CAO-affected testes in dogs, albeit in reduced numbers compared to normal canine control tissue [8,48], there is no information to date on the role of Sertoli cells in azoospermic dogs. The aim of this study was to verify the participation of Sertoli cells in canine CAO and contribute to the understanding of the development, maintenance and progression of canine CAO, also as a possible model for male infertility related to NOA, but also CAO and autoimmune orchitis. These investigations can form the basis for later therapeutic concepts to reinitiate the physiological environment by restoring Sertoli cell function and thus enable the “restart” of spermatogenesis with the help of the remaining SSCs.

## 2. Results

### 2.1. Expression of bFGF

**Figure 1:**
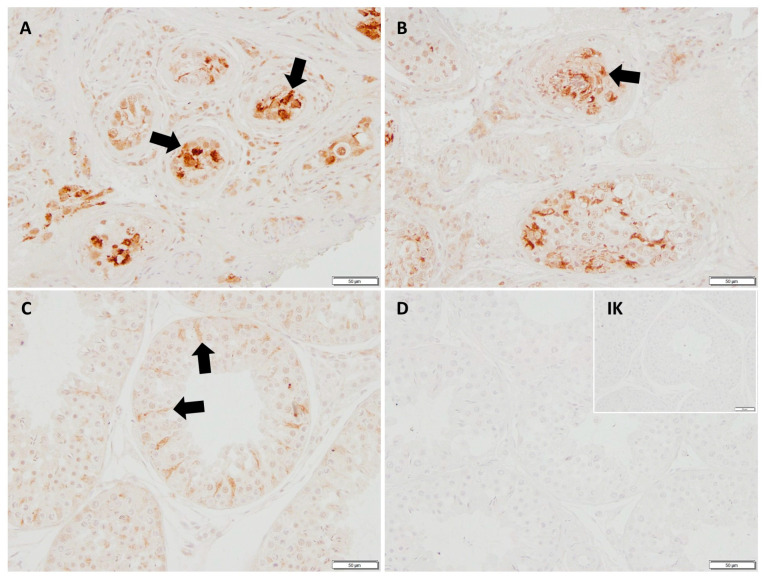
Immunostaining against bFGF (polyclonal rabbit anti-human antibody, Bs-2235r, Bioss, 20 µg/mL, 200× magnification), (**A**) chronic asymptomatic orchitis (CAO) early arrest, (**B**) CAO late arrest, (**C**) healthy control dog, (**D**) negative control and isotype control (IK; small insert, scale bar: 50 µm). A positive signal is visible in the Sertoli cell cytoplasm (black arrows) and some unspecific background staining.

The mRNA expression of *FGF2* revealed no significant differences between CAO-affected dogs and CG. Immunohistochemistry (IHC) revealed staining of the Sertoli cell cytoplasm of CAO and CG dogs; additionally, blood vessels were stained positive (Figure 1).

**Figure 2:**
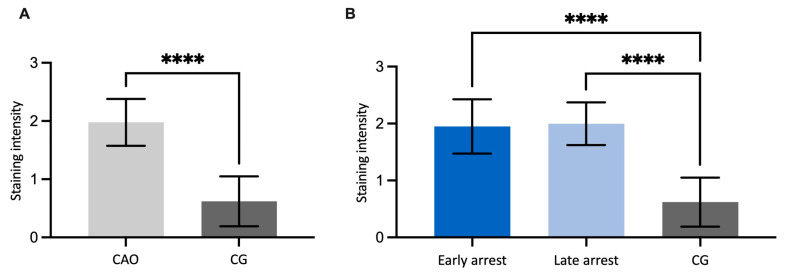
(**A**) bFGF protein expression evaluated by scoring the signal semi-quantitatively from 0 to 3 in 40 nearly round tubules per dog in chronic asymptomatic orchitis (CAO)-affected testis sample dogs compared to healthy control dogs (CG), and in (**B**), CAO is differentiated into early and late arrest. The results are presented as arithmetic mean and standard deviation. Datasets with asterisks differ significantly: each **** *p* < 0.0001.

A statistical evaluation of the bFGF IHC results disclosed significantly higher expression in CAO-affected testes (unpaired *t*-test, *p* < 0.0001, Figure 2A) compared to CG. Comparing staining intensity of CG to CAO early and late arrest, an overall significant difference was identified (ANOVA, *p* < 0.0001), with CG differing significantly from both CAO subgroups (Tukey’s multiple comparisons test, each *p* < 0.0001, Figure 2B). No significant difference was observed within the CAO group regarding early and late arrest.

**Figure 3:**
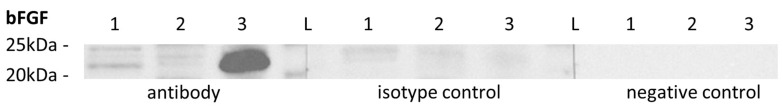
Western blot of bFGF using healthy control dog testis protein (lane 3). Protein size in kilodalton (kDa) is given on the left side. (1) indicates HeLa cell lysate, (2) rat testis, (3) healthy dog testis protein and (L) the ladder; antibody indicates that this part of the membrane was incubated with the respective bFGF antibody. The antibody was replaced by an irrelevant isotype control (“isotype control”) or buffer (“negative control”).

The Western blot revealed a specific band at around 22 kDa in the lane with the canine testicular protein of a healthy dog with normal spermatogenesis, confirming the specificity of the antibody (Figure 3). A faint band or no band was observable for HeLa cell lysate and rat testis. This is, however, not surprising, since Western blot was not specified as an application by the manufacturer of the antibody. Absent bands in the Western blot parts incubated in the isotype or negative control support the specificity of the bFGF antibody and its suitability for Western blots with canine samples.

### 2.2. Expression of GDNF

**Figure 4:**
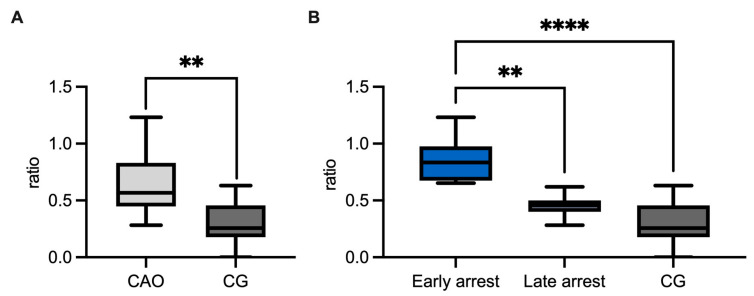
(**A**) *GDNF* mRNA expression (ratio) in dogs affected by chronic asymptomatic orchitis (CAO) and healthy controls (CG). (**B**) *GDNF* mRNA expression (ratio) in groups of early and late arrest (CAO) and control dogs (CG). The results are presented as Box and Whiskers. Datasets with asterisks differ significantly: ** *p* < 0.01, **** *p* < 0.0001.

Regarding the ratio (mRNA expression) of CAO and CG, qPCR demonstrated a significantly higher expression of *GDNF* in CAO compared to CG (unpaired *t*-test *p* = 0.0036, Figure 4A). Comparing the ratios of CG and CAO early and late arrest, an overall significant difference was identified (ANOVA, *p* < 0.0001). Tukey’s multiple comparisons test pointed out a significantly higher GDNF expression in the early arrest CAO samples compared to CG (*p* < 0.0001) and likewise compared to late arrest (*p* < 0.01), whereas the results of late arrest and CG did not differ significantly (Figure 4B).

**Figure 5:**
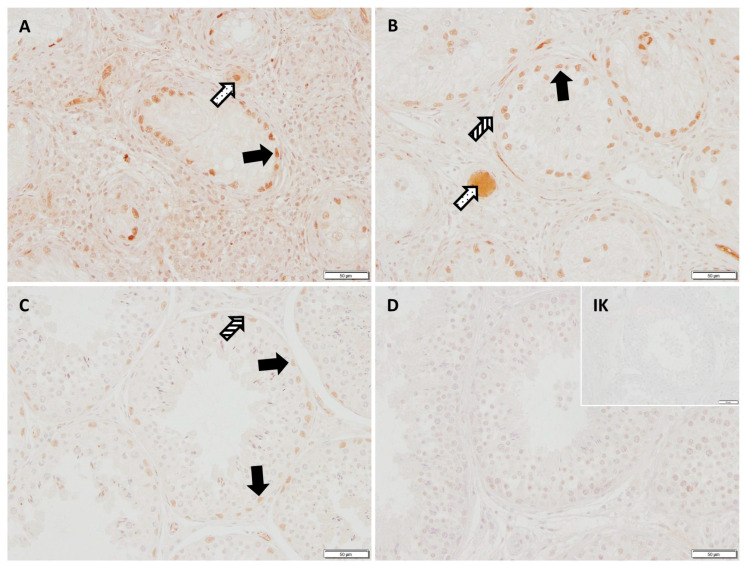
Immunostaining against GDNF (polyclonal rabbit anti-human antibody, orb1089007, Biorbyt, 2 µg/mL, 200× magnification), (**A**) chronic asymptomatic orchitis (CAO) early arrest, (**B**) CAO late arrest, (**C**) healthy control dog, (**D**) negative control and isotype control (IK; small insert, scale bar: 50 µm). Positive signals are visible in Sertoli cell nuclei (black arrows), blood vessels (dotted arrows) and peritubular myoid cells (striped arrows).

Immunopositive signals were observed in the nuclei of Sertoli cells, some peritubular cells, blood vessels and acrosomes in all dogs (Figure 5). A statistical analysis of scoring the proportion of stained nuclei revealed no differences in protein expression for GDNF in all groups.

**Figure 6:**
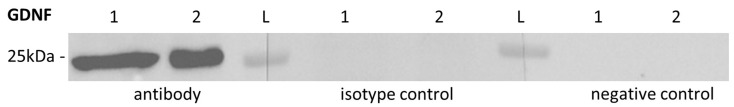
Western blot of GDNF. Rat testis served as a positive control; a testis homogenate of a healthy control dog was used. Protein size in kilodalton (kDa) is given on the left side. (1) indicates rat testis homogenate, (2) canine testis protein and (L) the ladder; antibody indicates that this part of the membrane was incubated with the respective GDNF antibody. The antibody was replaced by an irrelevant isotype control (“isotype control”) or buffer (“negative control”).

Western blot confirmed the specificity of the antibody used, showing a 24kDa protein band in dog and rat testis (positive control) (Figure 6). As for bFGF, absent bands in the isotype or negative control support specific antibody binding.

### 2.3. Expression of WNT5A

**Figure 7:**
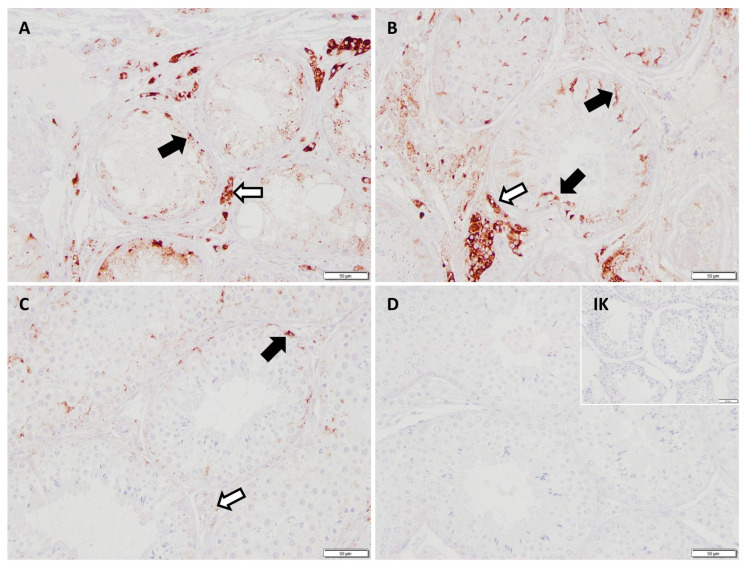
Immunostaining against WNT5a (polyclonal rabbit anti-human antibody, ab235966, Abcam, 1 µg/mL, 200× magnification), (**A**) chronic asymptomatic orchitis (CAO) early arrest, (**B**) CAO late arrest, (**C**) healthy control dog, (**D**) negative control and isotype control (IK; small insert, scale bar: 50 µm). An immunopositive signal is visible in the cytoplasm of Sertoli cells (black arrows) and Leydig cells (empty arrows).

Evaluation of the qPCR revealed no significant differences in the ratio of *WNT5A* between the groups. In the IHC of WNT5A, the cytoplasm of Sertoli cells as well as of Leydig cells were stained immunopositive in both CAO and CG dogs (Figure 7).

**Figure 8:**
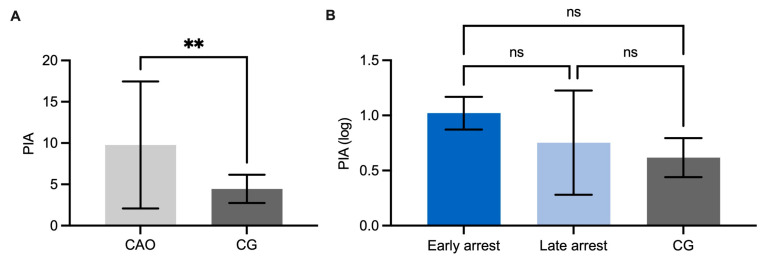
Evaluation of WNT5A protein expression regarding percentage of immunopositive area (PIA), (**A**) for overall group comparisons between chronic asymptomatic orchitis (CAO) and control group (CG), (**B**) CAO is divided into early and late arrest. The results are presented as arithmetic mean and standard deviation. Datasets with asterisks differ significantly: ** *p* < 0.01, ns = not significant.

Computer-assisted objective evaluation revealed a significantly higher PIA in CAO-affected dogs compared to CG (Mann–Whitney test, *p* = 0.0066) (Figure 8A), whereas an overall significance comparing PIA of CG and CAO early and late arrest by ANOVA could not be identified (Figure 8B).

The statistical evaluation of the mean grayscale revealed no significant differences between CAO and CG samples, indicating that staining intensity did not differ.

### 2.4. BMP4, CXCL12 and LDHC mRNA Expression

**Figure 9:**
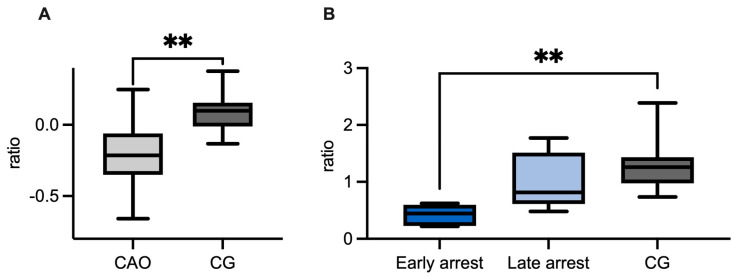
(**A**) *BMP4* mRNA expression (ratio) in chronic asymptomatic orchitis (CAO)-affected dogs and healthy controls (CG). (**B**) *BMP4* mRNA expression (ratio) in groups of early and late arrest (CAO) and control dogs (CG). The results are presented as Box and Whiskers. Datasets with asterisks differ significantly: ** *p* < 0.01.

**Figure 10:**
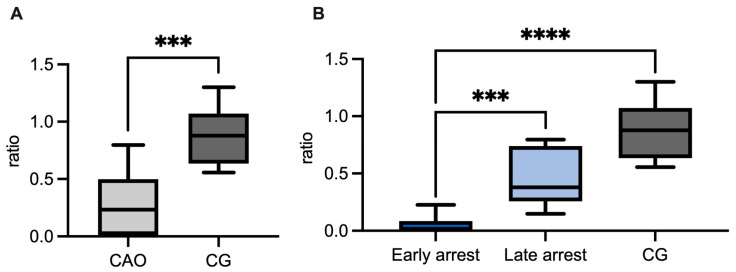
(**A**) *LDHC* mRNA expression (ratio) in (**A**) chronic asymptomatic orchitis (CAO) compared to healthy dogs (CG). (**B**) CAO divided into early arrest and late arrest compared to healthy control dogs (CG). The results are presented as Box and Whiskers. Datasets with asterisks differ significantly: *** *p* < 0.001, **** *p* < 0.0001.

*BMP4* and *LDHC* were significantly less expressed in CAO-affected testes compared to CG (unpaired *t*-test, *BMP4 p* = 0.0041; Mann–Whitney test, *LDHC p* = 0.0001) (Figure 9A and Figure 10A). Comparing ratios of CG and CAO early and late arrest, an overall significant difference was identified for *BMP4* (ANOVA, *p* = 0.0023), as well as *LDHC* (ANOVA, *p* < 0.0001). The *BMP4* Tukey’s multiple comparisons test showed significantly less expression in early arrest compared to CG (*p* < 0.01), but no significance regarding CG and late arrest, and late and early arrest (Figure 9B).

The ratio of *LDHC* gene expression was significantly lower in early arrest compared to CG (Tukey’s multiple comparisons test, *p* < 0.0001). Moreover, the difference between early and late arrest was also significant (higher expression in late arrest; Tukey’s multiple comparisons test *p* = 0.0001), but not between late arrest and CG (Figure 10B).

**Figure 11:**
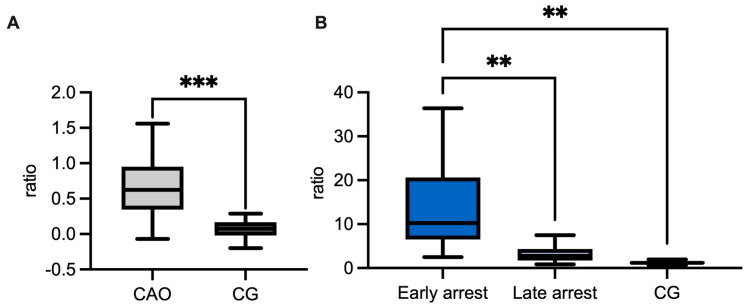
(**A**) *CXCL12* mRNA expression (ratio) in chronic asymptomatic orchitis (CAO) compared to healthy dogs (CG). (**B**) CAO divided into early arrest and late arrest compared to healthy control dogs (CG). The results are presented as Box and Whiskers. Datasets with asterisks differ significantly: ** *p* < 0.01, *** *p* < 0.001.

Both *BMP4* and *LDHC* were significantly downregulated in the early arrest CAO group. *CXCL12*, in contrast, showed a significant higher ratio in CAO compared to CG (Mann–Whitney test, *p* = 0.0003, Figure 11A). Comparing *CXCL12* ratios of CG and CAO early and late arrest, an overall significant difference was identified (ANOVA, *p* = 0.0016). Tukey’s multiple comparisons test ruled out significantly higher expressions of *CXCL12* in early arrest compared to late arrest samples and in the control group (each *p* < 0.01, Figure 11B). The difference between CG and late arrest was not statistically significant.

## 3. Discussion

The main cause of NOA [6] in acquired infertility was identified as CAO in male dogs [2], but also in men [3,4,49]. Unfortunately, no therapy options are available for both species, as reviewed by Vij et al. [50]. Knowing about the loss of SSC [8], further investigations about the functionality of Sertoli cells, the nourishing and supporting cells of germ cells, are required. The expression of various Sertoli cell-derived markers in the SSC niche in mice has been partially investigated and nicely reviewed by Mäkelä and Hobbs [21], but there are, especially in dogs, hardly any data. This study, for the first time, investigated the expression of different functional Sertoli cell markers (bFGF, BMP4, CXCL12, GDNF, LDHC and WNT5a) in healthy but also in CAO-affected canine testis.

Immunohistochemistry revealed the expression of bFGF, GDNF and WNT5A in different cellular compartments of the Sertoli cells but also other cells in canine testis. The protein expression of bFGF was visible in the cytoplasm of Sertoli cells. This growth factor is known to be essential for self-renewal [37,51] and maturation [52,53], underlining the strive of Sertoli cells in CAO testis to maintain or regain functionality. Different to dogs, rats [54] and cattle [55] might be able to better balance dysregulation via the additional expression of bFGF in Leydig and germ cells.

The indispensability of bFGF for SSC culturing is well established [24,56,57], but mostly together with GDNF [37,58,59], another important Sertoli cell-derived growth factor [26,60]. We identified GDNF in Sertoli cell nuclei, comparable to other species [26,61], and in some peritubular myoid cells as well, contributing to germ cell maintenance [62]. Whereas reduced GDNF and bFGF mRNA and protein expressions in human Sertoli cells have been published in the case of severely disrupted spermatogenesis, Sertoli-cell-only [63] and NOA [31] human patients, our study showed a trend toward upregulation of these factors, matching the findings of Jensen et al. in affected men [64]. Higher FSH levels led to the overexpression of bFGF in rats [53] and GDNF in human testis [65], as well as in Sertoli cell cultures from rats and mice [66,67,68,69]. Accordingly, the level of FSH concentration in peripheral blood is associated with the severity of spermatogenesis disorder in men [70]. Interestingly, the expression of GDNF and bFGF is particularly high in CAO samples with early arrest in our study. However, a possible connection between FSH, bFGF and GDNF in canine CAO remains speculative. As this study is solely based on leftover tissue from diagnostic biopsies and blood samples are not available, the FSH levels in our patient cohort cannot be measured at that point.

A higher GDNF expression caused the accumulation of undifferentiated spermatogonia in mice [26], while inhibiting GDNF caused the differentiation of SSC as a consequence [71], both not detectable in our CAO-affected testis. Additionally, the upregulation of PLZF (a Sertoli cell-independent factor for SSC self-renewal and maintenance) [7] and c-Kit mRNA expression [8] was described in SSC for the CAO-affected dogs and might be in response to the increased GDNF secretion by Sertoli cells, as proposed in mice [72]. Accordingly, a study with spermatogonial progenitor cells described increased levels of both GDNF-receptor and PLZF through inhibiting mTORC1 activity [73]. In contrast to GDNF, bFGF overexpression does not seem to be linked with spermatogonial differentiation in mice [74]. Nevertheless, as this is the first study detecting bFGF and GDNF, as well as the other investigated markers in canine testis, the conclusions drawn in rodent models warrant further verification for dogs.

The Sertoli cell factor *BMP4* is important for spermatogenesis by regulating SSC proliferation and differentiation [75,76]. In contrast to our findings, the *BMP4* ratio in human NOA patients was increased [64], whereas another study with men matching our results described lowered BMP4 expression in NOA, but only in comparison to obstructive azoospermia [31]. Due to the lack of specific canine antibodies, the observed modified *BMP4* gene expression could not be confirmed at the protein level. Different studies [21,77,78] promote GDNF as an antagonist to BMP4 regarding SSC differentiation. A shifted balance in favor of GDNF expression, as seen in our results, might thus inhibit SSC differentiation and lead to infertility. Furthermore, *BMP4*-knockout in sheep Sertoli cells resulted in decreased mRNA expression of the anti-apoptotic factor *Bcl-2* [77]. However, despite reduced *BMP4* levels, Morawietz et al. [17] observed increased Bcl-2 expression in the same set of canine CAO samples, rather supporting a theory of apoptosis resistance in Sertoli cells.

The WNT5A protein expression in immunohistochemistry was detected in Sertoli cell cytoplasm and Leydig cells. This expression pattern and the importance of WNT5A for spermatogenesis, promoting self-renewal of SSC [29] and regulating junctional function of Sertoli cells [79], were reviewed by Mei et al. [80]. However, information on WNT5A in dogs is lacking so far; thus, extrapolation from studies in mice and humans to dogs cannot be assured. Publications of transgenic mice revealed a connection of increased WNT signaling with testicular atrophy, tubulus degeneration and progressive germ cell loss, as well as the overexpression of AMH and GDNF [81,82]. This effect has also been observed in Connexin-43 knock-out mice with impaired BTB [83], whereas the alteration of CX43 expression is also known in our CAO-affected dogs [11]. An additional hint to the involvement of WNT5A in disturbed BTB is its impact through indirect mediation of the balance of mTORC1 and 2 [79]. The ratio (mRNA expression) of *WNT5A* did not to differ between CAO and CG; a true increase in Sertoli cell *WNT5A* expression might have been, however, masked because *WNT5A* expression is not restricted to Sertoli cells, but also in interstitial Leydig cells, and a relative increase in the interstitial compartment related to the CAO-associated pathohistological alterations was previously identified [6]. Tubule-/compartment-specific mRNA analysis using laser-assisted cell-picked tissue would be the best option to prove this hypothesis.

*CXCL12*, also known as stromal cell-derived factor 1 (SDF-1), expressed by Sertoli cells plays a crucial role in the maintenance of the spermatogonial population during postnatal development [32,84,85]. In adult testes, it is known to be involved in SSC propagation, preventing SSC differentiation together with its receptor CXCR4 [32]. The depletion of germ cells in mice led to the overexpression of *CXCL12* [86], matching our elevated mRNA expression results. In contrast, the interaction pair of CXCR4/CXCL12 was downregulated in idiopathic NOA of men [87]. Another interesting fact was that treatment with bFGF in TM4 mouse Sertoli cells increased *CXCL12* levels [88], which leads to the idea of a connection between the two Sertoli cell-derived factors.

Finally, *LDHC* expressions were studied with a significantly lower ratio in CAO compared to CG. *LDHC* is produced by germ cells and Sertoli cells in mice, humans and pigs [33,34,36]. The exact protein expression pattern in canine testes needs further investigations. LDHC, as terminal enzyme for glycolysis, is required for sperm motility, as it provides pyruvate as the preferred energy source, and therefore male fertility in mice [35]. The lower ratio of *LDHC* in early arrest CAO can be explained by the massive decrease in germ cells in the tubules, as its expression is not limited to Sertoli cells or may only be expressed by germ cells, as some studies indicate [89]. Nevertheless, a recent study of biomarkers in human seminal plasma identified a LDHC decrease in NOA-affected patients [90]. The mRNA results clearly underline that the severity of tissue damage was in line with the extent of up- (*GDNF* and *CXCL12*) and downregulation (*BMP4* and *LDHC*) of Sertoli cell markers. Thus, biomarker analysis in seminal plasma might be a good target for future non-invasive, more detailed diagnostics of CAO in dogs, too.

Although we cannot draw conclusions about Sertoli cells from *LDHC* ratios, the theory of immature Sertoli cells in CAO testes in dogs is supported by AMH expression (unpublished data) and WNT5A upregulation, as the inhibition of WNT signaling promotes Sertoli cell maturation [39]. Lower BMP4 levels in pre-pubertal Tibetan sheep compared with adult testes, matching our observation in CAO testes, further support Sertoli cell immaturity [77]. Additionally, GDNF [91,92] and bFGF [93] have proliferative effects on Sertoli cells, and Sertoli cells are known to proliferate in an immature state during minipuberty. Coculturing of healthy, mature Sertoli cells with the testicular tissue of NOA-affected testis in human has not yet been successful but sets a good starting point for future, comprehensive therapeutic options [94].

## 4. Materials and Methods

### 4.1. Study Populations and Tissue Collection

This study includes 25 clinically healthy, sexually matured dogs. All samples were used with owners’ consent. In the control group (CG) (n = 10), testicular tissues were obtained by castration at the owner’s request and, in the azoospermic group (n = 15), by biopsy for diagnostic purposes to identify the underlying cause of infertility and possible treatability. All dogs underwent clinical examinations to confirm that they were clinically healthy. Moreover, semen collection was performed in the presence of a teaser bitch. Dogs presented for surgical castration were included in CG in the case of normospermia. For azoospermic dogs, repeated semen collections within 3 months apart were performed, revealing azoospermia or one semen collection with an azoospermic sample combined with a prolonged history of infertility. As previously described [6], an andrological examination revealed softer testes of the affected dogs. An analysis of the Alkaline Phosphatase in the seminal plasma of the second fraction of the ejaculate confirmed non-obstructive azoospermia and ruled out obstructive azoospermia. A conventional aerobic bacteriological culture ruled out bacterial infection and endocrinological testing (LH, testosterone, estradiol, thyroidal hormones and anti-thyroglobulin antibodies) endocrine abnormalities. Samples of CG and the azoospermic group were obtained under general anesthesia, and all dogs received carprofen (4 mg/kg BW) orally for three days in CG and five days in the azoospermic biopsy group. All testicular tissue samples were processed as previously described [48,95,96]. Briefly, one part was placed in RNAlater^®^ (Qiagen GmbH, Hilden, Germany) and frozen at−80 °C for subsequent mRNA and protein extraction, whereas another part was fixed in Bouin’s solution for 24h and washed several times in 70% ethanol before paraffin embedding for histology. All included azoospermic dogs were diagnosed with chronic asymptomatic orchitis (CAO) defined by significant immune cell infiltration and disruption of spermatogenesis [6]. According to the histological findings, this group was divided into two subgroups: 1. early arrest, including Sertoli cells only or spermatogenesis arrested at the level of spermatogonia (n = 6), and 2. late arrest, with spermatogenesis arrested at the level of spermatocytes or later stages (n = 9) [8,17].

The average age of the CAO group was 5.5 ± 1.9 years (2.5–9.5 years), and the dogs were of the following breeds: n = 1 each: Beagle, Cairn Terrier, Cane Corso, Coton de Tulear, Iceland Sheepdog, Jack Russel Terrier, Labrador Retriever, Miniature Poodle, Welsh Corgi Pembroke; n = 3: Collie, German Shepherd Dog. The control group (CG) had an average age of 3.8 ± 3 years (0.9–9.9 years) and included the following breeds: n = 1 each: Boston Terrier, Boxer, Bernese Mountain dog/Newfoundland Crossbreed, Chihuahua, Havanese, Maltese, Mongrel; n = 3: Beagle.

### 4.2. Quantitative Real-Time PCR

The RNA samples and cDNA were prepared as described before [8]. The RNA was isolated using TRI reagent^®^ (T9424, Sigma Aldrich, St. Louis, MO, USA), concentration measured using a NanoPhotometer^®^ NP80 (IMPLEN, Munich, Germany), and cDNA synthesized using 200 ng/µL RNA and RevertAid First Strand cDNA Synthesis kit (#K1622, Thermo Fisher, Waltham, MA, USA). To measure the expression of *FGF2* (encoding for bFGF), *BMP4*, *CXCL12*, *GDNF*, *LDHC* and *WNT5A*, primer sets for RT-qPCR were drafted using BLAST (https://blast.ncbi.nlm.nih.gov/Blast.cgi, accessed on 19 February 2024) (Table 1). PCR product sequencing (Microsynth AG, Balgach, Switzerland) confirmed specific primer binding. For endogenous control, *GAPDH* (Glyceraldehyde-3 phosphatase dehydrogenase) and *HPRT* (hypoxanthine guanine phosphoribosyltransferase) served as reference genes.

A relative mRNA expression (RT-qPCR) analysis was performed as previously described [17,97]: 2 µL of 1:10 diluted cDNA was added to 8 µL of FastStart Essential DNA Green Master (Roche Diagnostics GmbH, Mannheim, Germany), 1 µL of the forward and reverse primer (10 pmol) (Table 2), and 2 µL of sterile Aqua bidest. A LightCycler^®^ real-time PCR system (Software version 1.1.0.1320, Roche Diagnostics GmbH, Mannheim, Germany) was used for all samples. The following cycling conditions were used in RT-qPCR for all genes: 95 °C for 10 min, followed by 35 cycles of 95 °C for 10 s, 60 °C for 10 s, 72 °C for 10 s, and a melting curve. All samples were run in triplicates. *GAPDH* and *HPRT* were chosen as reference genes, as they were previously determined to be most stably expressed in this specific sample set and superior to *ACTB* [8]. For the calculation of PCR efficiencies, a relative standard curve derived from a triplet RT-qPCR run of a 2-fold dilution series (1:2–1:128) of pooled cDNA samples was used. To evaluate the RT-qPCR results, the efficiency-corrected relative quantification according to Pfaffl (2001) [98] was modified and extended, taking both reference genes into account as described previously [99]. BLAST (https://blast.ncbi.nlm.nih.gov/Blast.cgi, accessed on 7 March 2024) was used to verify the specificity of primers for *FGF2*, *BMP4*, *CXCL12*, *GDNF*, *LDHC* and *WNT5A* used in RT-qPCR, and the results were confirmed by sequencing PCR products (Microsynth AG, Balgach, Switzerland).

### 4.3. Immunohistochemistry

IHC was performed as described previously [8,100]. Briefly, Bouin-fixed sections were deparaffinized, and antigen retrieval was performed via heating until boiling in a citrate buffer (pH 6.0). After quenching the endogenous peroxidase reactivity by 3% hydrogen peroxide in methanol, the unspecific bindings were blocked with 3% bovine serum albumin (BSA, VWR Life Science, Solon, OH, USA) diluted in 10% goat serum (S-2000, Vector Laboratories, Newark, CA, USA). Incubation was performed overnight at 4 °C with the primary antibodies and dilutions listed in Table 3. Each step was followed by washing with ICC buffer (1.2 g Na_2_HPO_4_, 0.2 g KH_2_PO_4_, 0.2 g KCl, 8.0 g NaCl, 3 mL Triton ad 1000 mL) three times. ICC buffer served as a negative control; irrelevant rabbit IgG (I-1000, Vector Laboratories) served as an isotype control diluted in the same concentration as the primary antibody. Afterwards, sections were incubated at room temperature for 30 min with biotinylated goat anti-rabbit secondary antibody (BA-1000, Vector Laboratories, dilution 1:200). Visualization was performed with the avidin-biotin-peroxidase complex (ABC) procedure using a commercial immunoperoxidase kit (VECTASTAIN PK-6101 Elite ABC Kit, Vector Laboratories) and NOVA red (Vector Nova-RED Substrate Kit SK-4800, Vector Laboratories). All sections except from WNT5A were counterstained with Meyer’s hematoxylin, dehydrated and mounted in Roti^®^ Histokitt II (Roth AG, Arlesheim, Switzerland) for microscopic examination. Evaluation of the IHC staining for bFGF, GDNF and WNT5A was carried out by using an Olympus BX41TF Microscope (Olympus^®^, Tokyo, Japan) with an Olympus DP72 camera (Olympus Corporation, Tokyo, Japan) and the Olympus cellSense Dimension Software (version 2.1, Olympus Corporation, Tokyo, Japan). Forty nearly round tubules of each dog (CAO: 20 from each side) were evaluated at 200-fold magnification with semiquantitative ranking (0–3) of the positive signal. In bFGF staining, intensity was ranked from 0 (no staining) to 3 (highest intensity). GDNF was counted from 0 (no Sertoli cell nuclei are stained) to 3 (all SC nuclei stained). The computer-assisted evaluation of tubular WNT5A staining (40 tubules at 200-fold magnification) using GIMP (GNU Image Manipulation Program 2.10.36, open source, Mountain View, CA, USA, https://www.gimp.org/) and ImageJ (ImageJ 1.54g, public domain, Bethesda, MD, USA, https://imagej.net/ij/index.html, accessed on 23 January 2024) resulted in the percentage of the immunopositive area (PIA) as well as the staining intensity (mean grayscale).

### 4.4. Western Blot

A Western blot analysis was performed following verification of the specificity of the primary antibodies used in the IHC. Preparation of the dogs’ protein (approximately 0.8 g) was described earlier [8]. After denaturation of the protein for ten minutes in the water bath at 96 °C, separation of the protein and Precision Plus Protein™ All Blue Prestained Protein Standards (Bio-Rad Laboratories) as the ladder was carried out via gel electrophoresis using 4–20% gradient sodium dodecyl sulfate-polyacrylamide gel (Mini-Protean^®^ TGXTM Gels, Bio-Rad Laboratories, Hercules, CA, USA). The Trans-Blot^®^ TurboTM Transfer Pack (Bio-Rad Laboratories) was used to transfer the protein onto a PVDF membrane (Immuno-Blot^®^ PVDF Membrane, Bio-Rad Laboratories); then, blocking was performed for five minutes (EveryBlot Blocking Buffer, Bio-Rad Laboratories). After this step, the membranes were cut into three parts, leaving a complete lane of the positive control and canine testes in each part. The first part was incubated with primary antibody (bFGF in dilution 1:200, GDNF in 1:500), the second part with irrelevant rabbit IgG (I-1000, Rabbit IgG, Control Antibody, Vector Laboratories) serving as the isotype control and the third part in TBS as the negative control, all overnight at 4 °C. Apart from this step, all three parts were treated equally in the following. After washing with TBS (tris-buffered saline), secondary antibody (Goat Anti-Rabbit, Peroxidase, PI-100-1, Vector Laboratories) at a dilution of 1:1000 was added for one hour. After another round of washing, the membrane parts were reassembled, signals were visualized using Clarity™Western ECL Blotting Substrate (Bio-Rad Laboratories) for one minute, images were taken with ChemiDoc™ Imaging Systems with Image Lab™ Touch Software (Image Lab 6.0.1, Bio-Rad Laboratories) and band size was identified. Correct bands in the antibody-incubated part, but not in the isotype or negative control, indicated specific antibody binding. The WNT5A antibody was not suitable for WB. For bFGF, the HeLa cell lysate served as a positive control, and for GDNF, rat testis.

### 4.5. Statistical Analysis

The aim of this study was to analyze significant differences in the mRNA expression between CAO and CG for the functional Sertoli cell markers *FGF2* (*bFGF*), *BMP4*, *CXCL12*, *GDNF*, *LDHC* and *WNT5A*. At the level of protein expression, bFGF, GDNF and WNT5A were compared. GraphPad Prism10 software (GraphPad Software, Inc., La Jolla, CA, USA) and Microsoft Excel (Version 16.81, Microsoft, Redmond, WA, USA) were used; values at a level of *p* < 0.05 were rated as statistically significant.

To compare the results of protein expression, all data were tested by the Shapiro–Wilk test for normal distribution, which could be confirmed for GDNF, bFGF and mean grayscale of WNT5A. The paired *t*-test was applied to rule out differences between the right and left testes in the CAO group, which was not given, so the results from both sides of each dog were summarized. For the group comparisons, ordinary one-way ANOVA was used, followed by Tukey’s multiple comparisons test if *p* < 0.05 for further details. An unpaired *t*-test was performed for a general comparison between CAO and CG. Since the data of WNT5A PIA within the detailed group division were only log-normally distributed after applying the Shapiro–Wilk test, they were transformed and then compared using ordinary one-way ANOVA. For a comparison of CAO and CG, the Shapiro–Wilk test revealed no normal distribution so the Mann–Whitney test was used. Protein data are presented as arithmetic mean and standard deviation for comparative reasons.

The results of the mRNA expression were also tested by the Shapiro–Wilk test for normal distribution. As a result of *BMP4* and *CXCL12* being normally distributed, ordinary one-way ANOVA could be used directly for these two, while *FGF2*, *GDNF* and *LDHC* had to be log-transformed before. Afterwards, Tukey’s multiple comparisons test was used for further details if *p* < 0.05. As the PCR data of *WNT5A* were not normally distributed, the Kruskal–Wallis test was applied, followed by Dunn’s multiple comparison test if *p* < 0.05 for further details. For log-normally distributed markers *bFGF*, *BMP4*, *CXCl12* and *GDNF*, an unpaired *t*-test and, for *LDHC* and *WNT5A*, the Mann–Whitney test were performed for an overall comparison between CAO and CG. Data of the mRNA expression are presented as Box and Whisker Plots with their median, maximum and minimum.

## 5. Conclusions

This study not only reveals new insights into the pathophysiology of NOA and CAO in dogs, but it also provides first insights into the Sertoli cell function of dogs in general. The dysregulation of the functional Sertoli cell markers GDNF, bFGF, BMP4, CXCL12, LDHC and WNT5A, depending on the severity of histological changes in the CAO-affected testis, reveals further aspects of the interactions in the SSC niche and illustrates the importance of the maturation status of Sertoli cells. We were not able to rule out the exact cause of CAO, but this research shows further correlations of already researched changes in CAO-affected dogs, regarding changes in the BTB, and maturation status, which might help us to get closer to an NOA treatment. Based on this knowledge, pure SSC-based therapy without optimization of the Sertoli cells and SSC microenvironment is likely not successful.

## Figures and Tables

**Table 1:** Investigated Sertoli cell-derived factors, with protein and gene symbol and function.

Sertoli Cell-Derived Factor	Protein (*Gene*) Symbol	Function	Reference
basic fibroblast growth factor	bFGF (*FGF2*)	bFGF and leukemia inhibitory factor (LIF) promote proliferation of human SSCs in short-term Sertoli germ cell co-culture	[22,23,24]
glia cell line-derived neurotrophic factor	GDNF (*GDNF*)	Undifferentiated spermatogonia convert between stem cells and progenitor cells, based on the presence of GDNF in mice	[23,25,26]
Wnt oncogene analog 5a	WNT5A (*WNT5A*)	Androgen-regulated Sertoli cell gene, involved in the precisely controlled regulation of SSC self-renewal; inhibition of WNT signaling promotes Sertoli cell maturation in mice	[27,28,29]
bone morphogenetic protein 4	BMP4 (*BMP4*)	Influences Sertoli cell proliferation and DNA synthesis and directs the differentiation of spermatogonia in mice; downregulated in NOA-affected men	[30,31]
C-X-C motif chemokine ligand 12	CXCL12 (*CXCL12*)	Regulation of self-renewal and maintenance of SSC; blockage of the CXCL12-CXCR4 signaling pathway leads to SSC death in mice	[23,32]
lactate dehydrogenase C	LDHC (*LDHC*)	Testis-specific, produced post-pubertally by germ cells and Sertoli cells; LDHC-deficient male mice are infertile; LDHC-expressing Sertoli cells provide lactate as energy resource for spermatocytes and spermatids in pigs	[33,34,35,36]

**Table 2:** Sequences of primers for RT-PCR and RT-qPCR, amplicon length, efficiency and accession number (for, forward; rev, reverse).

Primer	Oligonucleotide Sequence (5′-3′)	Amplicon Length (bp)	Efficiency	Accession Number
*GAPDH*		228	2.05	NM 001003142
for	GGCCAAGAGGGTCATCATCTC			
rev	GGGGCCGTCCACGGTCTTCT			
*HPRT*		94	2.05	NM 001003357.2
for	TGACACTGGGAAAACAATGCA			
rev	GGTCCTTTTCACCAGCAAGCT			
*FGF2*		148	2.08	XM 038565228.1
for	ACCTTCCTTTCACACTCCACCC			
rev	TGTTTCCCTCCACTGTTTCATTCA			
*BMP4*		216	1.90	NM 001287170.2
for	TTGATGTGAGCCCTGCGGTC			
rev	GTCGGGTCAAGGCATGTCCG			
*CXCL12*		142	2.12	NM 001128097.1
for	ACTCCGAACTGTGCCCTTCA			
rev	CCACCTGCGCCTCTCACAT			
*GDNF*		175	2.11	XM_038663888.1
for	CGCCTACGATGACGACCTGT			
rev	CGGGGCAAACATTTCCTGGG			
*LDHC*		92	1.98	NM 001271792.1
for	ACCATTGTTGGAACTGGTGCTG			
rev	GCAACATCAACGAGCGCAAG			
*WNT5A*		198	2.16	NM 001287075.1
for	TTCTCCTTCGCCCAGGTTGT			
rev	CGTCTTTGCGCCTTCTCCAA			

**Table 3:** Overview of reagents and dilutions used in the IHC.

Antibody	SKU	Source	Clonality	Dilution	Concentration (µg/mL)	Positive Control
bFGF	Bs-2235r *	rabbit anti-human	polyclonal	1:50	20	Rat brain
GDNF	orb1089007 **	rabbit anti-human	polyclonal	1:100	2	Rat dorsal root ganglion
WNT5A	ab235966 ***	rabbit anti-human	polyclonal	1:500	1	Human tonsil

* Bioss Antibodies, Woburn, MA, USA, ** Biorbyt, Cambridge, UK, *** Abcam, Cambridge, UK.

## Data Availability

The data presented in this study and supporting the conclusion of this research are available on request from the authors, without undue reservation.
